# Clinical and biochemical heterogeneity between patients with glycogen storage disease type IA: the added value of CUSUM for metabolic control

**DOI:** 10.1007/s10545-017-0039-1

**Published:** 2017-04-10

**Authors:** Fabian Peeks, Thomas A. H. Steunenberg, Foekje de Boer, M. Estela Rubio-Gozalbo, Monique Williams, Rob Burghard, Fabienne Rajas, Maaike H. Oosterveer, David A. Weinstein, Terry G. J. Derks

**Affiliations:** 1 Section of Metabolic Diseases, Beatrix Children’s Hospital, University Medical Center Groningen, University of Groningen, PO Box 30 001, Groningen, 9700 RB The Netherlands; 2 Department of Pediatrics, Center for Liver Digestive and Metabolic Diseases, University Medical Center Groningen, University of Groningen, Groningen, the Netherlands; 3 Department of Pediatrics, Maastricht University Medical Center, Maastricht, the Netherlands; Laboratory of Genetic Metabolic Diseases, Maastricht University Medical Center, Maastricht, the Netherlands; 4 Erasmus MC-Sophia Kinderziekenhuis, Erasmus Universiteit Rotterdam, Rotterdam, Netherlands; 5 EnerGQcare BV, Groningen, the Netherlands; 6 Institut national de la santé et de la recherche médicale U1213, Université Lyon 1, Lyon, France; 7 Glycogen Storage Disease Program, University of Connecticut School of Medicine and Connecticut Children’s Medical Center, Hartford, CT USA

**Keywords:** CUSUM, ESGSDI, GSD Ia, *G6PC*, Heterogeneity, Modifying factors

## Abstract

**Objective:**

To study heterogeneity between patients with glycogen storage disease type Ia (GSD Ia), a rare inherited disorder of carbohydrate metabolism caused by the deficiency of glucose-6-phosphatase (G6Pase).

**Study design:**

Descriptive retrospective study of longitudinal clinical and biochemical data and long-term complications in 20 GSD Ia patients. We included 11 patients with homozygous *G6PC* mutations and siblings from four families carrying identical *G6PC* genotypes. To display subtle variations for repeated triglyceride measurements with respect to time for individual patients, CUSUM-analysis graphs were constructed.

**Results:**

Patients with different homozygous *G6PC* mutations showed important differences in height, BMI, and biochemical parameters (i.e., lactate, uric acid, triglyceride, and cholesterol concentrations). Furthermore, CUSUM-analysis predicts and displays subtle changes in longitudinal blood triglyceride concentrations. Siblings in families also displayed important differences in biochemical parameters (i.e., lactate, uric acid, triglycerides, and cholesterol concentrations) and long-term complications (i.e., liver adenomas, nephropathy, and osteopenia/osteoporosis).

**Conclusions:**

Differences between GSD Ia patients reflect large clinical and biochemical heterogeneity. Heterogeneity between GSD Ia patients with homozygous *G6PC* mutations indicate an important role of the *G6PC* genotype/mutations. Differences between affected siblings suggest an additional role (genetic and/or environmental) of modifying factors defining the GSD Ia phenotype. CUSUM-analysis can facilitate single-patient monitoring of metabolic control and future application of this method may improve precision medicine for patients both with GSD and remaining inherited metabolic diseases.

## Introduction

Glycogen storage disease type Ia (GSD Ia; OMIM #232200) is a rare inherited disorder of carbohydrate metabolism caused by mutations in the *G6PC* gene, resulting in deficiency of glucose-6-phosphatase (G6Pase). The subsequently impaired hydrolysis of glucose-6-phosphate (G6P) to glucose and phosphate affects the final common pathway of glycogenolysis and gluconeogenesis (Bali et al n.d.; Froissart et al 2011). Symptoms and signs include severe fasting intolerance, failure to thrive, and hepatomegaly. Biochemically, the phenotype is characterized by non-ketotic hypoglycemia, hyperlactidemia, hyperuricaemia, and hyperlipidaemia (Bali et al n.d.). Dietary management has greatly improved the life expectancy of GSD Ia patients, changing from an acute, fatal disease into a chronic disorder. Despite intensive dietary management, important long-term complications include the liver (hepatocellular adenomas and carcinomas), kidneys (proteinuria, renal insufficiency, stones), and bone (osteopenia, osteoporosis) (Bali et al n.d.; Rake et al 2002a, b).

Cross-sectional studies such as the European Study on Glycogen Storage Disease Type 1 (ESGSDI) focused on the *complete* cohort of GSD Ia patients, but longitudinal data on clinical heterogeneity *between individual* GSD Ia patients have been poorly documented. In contrast with the classical childhood GSD Ia phenotype, case reports illustrate patients with milder phenotypes, clinically presenting during late childhood with non-symptomatic hepatomegaly or adulthood with gouty arthritis and benign/malignant hepatic tumors (Takahashi et al 2000; Shieh et al 2011; Cassiman et al 2010; Nakamura et al 2001; Matern et al 2002; Keller et al 1998). Although these patients have not experienced clinically relevant fasting intolerance, their abnormal biochemical profiles resemble classical GSD Ia patients. In addition, observations in two siblings suggest that clinical heterogeneity cannot be solely explained by the *G6PC* genotype (Rake et al 2000a). Furthermore, data analysis has focused largely on traditional methods describing differences between groups by expressing means or medians. However, patient care for metabolic patients often is characterized by repeated clinical and biochemical measurements and their analysis can be complemented by inter-individual analysis methods, such as Cumulative Sum analysis (CUSUM-analysis).

This is a retrospective study of longitudinal clinical and biochemical parameters from (1) GSD Ia patients with homozygosity for different *G6PC* mutations and (2) patients within GSD Ia families carrying identical *G6PC* genotypes.

## Patients and methods

### Patients

The Medical Ethical Committee of the University Medical Center Groningen approved the study protocol (MEC 2014|342). Data were studied from GSD Ia patients followed by two centers. Patients were selected based on *G6PC* genotypes/mutations and the availability of sufficient data. For all GSD Ia patients in this study the diagnosis was genetically confirmed and displayed according to the reference sequence NM_000151.3.

### Clinical and biochemical data

Longitudinal data on clinical and laboratory data and long-term complications were retrieved from the paper and electronic files before 01–02-2016.

Clinical parameters included height, weight, weight for height, BMI, and data of the prescribed diets. Height and BMI were recorded at last check-up and compared with Dutch standard growth diagrams (LUMC-TNO 1997 in cases A, B, and C and families I-III; LUMC-TNO 2010 in case D). For the patients from the University of Florida, biometric data were compared to the standard growth diagrams from the CDC 2000. Target height range was determined accordingly for all patients.

Biochemical parameters included blood concentrations that are closely related to metabolic control (i.e., lactate, uric acid, triglycerides (TG), and cholesterol) and urine parameters (i.e., creatinine, albumin and total protein) as mentioned in the published guidelines(Rake et al 2002a; Kishnani et al 2014).

Long-term complications were recorded at the last check-up. Liver adenoma(s) was defined as one or more focal lesions detected by standard imaging techniques. Nephropathy was defined as micro albuminuria (either 30–300 mg/24 h, or if previous data was not available albumin/creatinine >3.5 and >2.5 for females and males, respectively) and/or proteinuria (protein/creatinine >45 mg/mmol). Bone mineral density was evaluated by duel-energy X-ray absorptiometry scan (DEXA). Osteopenia was defined as bone mineral density T-scores between −1.0 and −2.5 SDs determined at one site. Osteoporosis was defined as bone mineral density T-scores of −2.5 SDs or lower determined at one site. The values are compared to the ideal or peak bone mineral density of healthy 30-year old adults.

### Statistical analysis

Statistical analysis was performed using Microsoft® Excel for Mac Version 15.19.1 and Graphpad Prism version 5.03 for Windows (San Diego, CA, USA, (www.graphpad.com)). Differences between groups were studied using either Mann-Whitney U test (in families I, II and IV) or Kruskal-Wallis test followed by Dunn’s multiple comparison (in patients with homozygous *G6PC* mutations and family III). Differences were considered statistically significant at *p* < 0.05.

To display subtle variations for repeated measurements with respect to time for individual patients, CUSUM-analysis graphs were constructed. CUSUM-analysis is a method in which each measurement is seen as a deviation from the mean value of the parameter over time. The cumulative effect of the deviations of each measurement to the mean is made visible as CUSUM-analysis graphs. However, in our retrospective analysis, interpretation of CUSUM-analysis was complicated because time intervals between TG measurements were not constant, which means that periods of high measurement density would have a disproportional effect in the CUSUM-analysis. To correct for different time intervals, the TG values were interpolated to equidistant time intervals (*t* = 0.01 year, approximately 3.65 days). This interpolation interval was chosen to make the calculation of the CUSUM easier. After calculating average blood TG concentrations (TG_mean_), for each value ∆TG was calculated as TG_n_-TG_mean_. At the first time point CUSUM equals ∆TG. For serial measurements at time point n, CUSUM is calculated as ∆TG + CUSUM_n-1_.

## Results

Twenty GSD Ia patients were included from 14 families, 12 males and eight females. Median age was 21.5 years (range 4.2–43.0).

### Differences between GSD Ia patients with homozygosity for different G6PC mutations

**Fig. 1:**
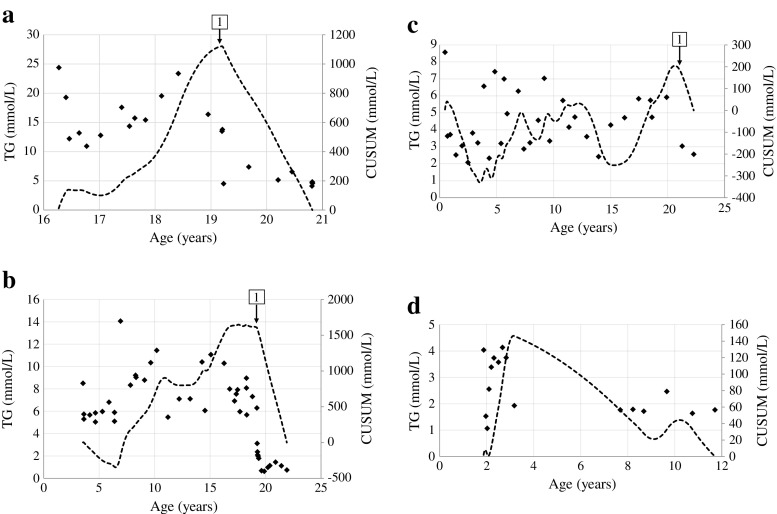
Longitudinal data of blood triglyceride concentrations (*diamonds*) and the CUSUM-analysis (*dashed line*) for patient A (**a**), patient B (**b**), patient C (**c**), and patient D (**d**). The arrows are explained in the text

Parameters of 11 patients with homozygosity for different *G6PC* mutations are presented in Table 1 (UMCG; patients A-D) and Table 2 (UF; patients E-K). Figure 1 presents (1) longitudinal data of blood TG concentrations and the first order derivative of blood TG concentrations with respect to time and (2) CUSUM-analysis for patients A-D.

**Table 1:** Clinical and biochemical parameters in four GSD Ia patients with homozygosity for one *G6PC* mutation, who are followed in the UMCG

Case	A	B	C	D
*G6PC* mutation				
*cDNA*	c.79delC	c.247C > T	c.467G > T	c.1039 C > T
*protein*	p.Gln27Argfs*9	p.Arg83Cys	p.Trp156Leu	p.Gln347X
Descent	Caucasian	Turkish	Caucasian	Caucasian
Gender	Female	Female	Male	Female
Year of birth	1994	1994	1992	2003
Age at clinical presentation (months)	0	2	0	22
Largest height				
(cm)	150	172	176	151
(SDS)	−3.3^	+0.3	−1.2	−0.4
BMI				
(kg/m2)	26.6	27.7	19.7	17.2
(SDS)	+1.6	+2.0	−0.9	−0.1
Lactate (mmol/L)	5.8^c,d^ (2.3–10.6)	4.3^c,d^ (0.9–18.6)	2.7^a,b^ (0.8–5.5)	1.4^a,b^ (1.0–1.4)
Uric acid (mmol/L)	0.28 (0.12–0.46)	0.29 (0.16–0.58)	0.26 (0.16–0.38)	0.28 (0.25–0.33)
Triglycerides (mmol/L)	12.5 (0.2–24.4)	6.1 (0.6–14.1)	4.2 (2.1–8.6)	2.2 (1.1–4.1)
Cholesterol (mmol/L)	10.3 (6.8–14.5)	5.0 (3.2–6.3)	5.9 (3.3–7.6)	3.6 (2.5–5.1)
Liver adenoma(s)	Yes	Yes	No	No
Nephropathy	No	No	No	No
Bone disease				
*Osteoporosis*	LS	LS,PF,R	LS	No
*Osteopenia*	No	No	No	LS, PF, R

Legend: Biochemical parameters are presented as median and range. Patient C corresponds with patient III.7 in Table 3. ^a^, significantly different compared to case A, ^b^, significantly different to case B, etc.; ^, height outside of target range; LS, lumbar spine; NR, not recorded; PF, proximal femur; R, radius. Differences were considered statistically significant at *p* < 0.05

**Table 2:** Clinical and biochemical parameters in seven GSD Ia patients with homozygosity for one *G6PC* mutation, who are followed in the GSD program, University of Florida

Case	E	F	G	H	I	J	K
*G6PC* mutation							
*cDNA*	c.247C > T	c.79delC	c.379_380 dupTA	c.467G > T	c.79C > T	c.379_380 dupTA	c.323C > T
*protein*	p.R83C	p.Gln27Argfs*9	p.Y128Tfs	p.W156 L	p.Q27X	p.Y128Tfs	p.T108I
Descent	Caucasian	Caucasian	Hispanic	Caucasian	Indian	Hispanic	Lebanese
Gender	Female	Male	Female	Male	Female	Male	Female
Year of birth	1983	1994	2011	2002	2000	2007	1997
Age at clinical presentation (months)	5	2	0	91	0	0	12
Last measured height							
(cm)	152.4	178.6	93.1	144.0	140.0	126.4	158.3
(SDS)	−1.7^	0.2	0.0	−0.6	−1.1	−1.0	−0.8
BMI							
(kg/m2)	25.1	25.6	17.4	20.5	21.2	20.2	40.2
(SDS)	NR	NR	NR	NR	NR	NR	NR
Lactate (mmol/L)	4.1^f,g,h,i,j,k^ (0.5–10.9)	1.9^e,h,i,j^ (0.7–4.8)	1.6^e^ (1.4–1.9)	1.2^e,f,j^ (0.6–3.2)	1.3^e,f^ (0.9–4.5)	1.5^e,h^ (0.7–5.1)	1.6^e,f^ (0.3–3.3)
Uric acid (mmol/L)	0.37^k^ (0.21–0.58)	0.44^i^ (0.32–0.55)	0.32^k^ (0.24–0.39)	0.40^k^ (0.29–0.50)	0.28^f,k^ (0.21–0.37)	0.29^k^ (0.25–0.58)	0.52^e,g,h,i,j^ (0.42–0.65)
Triglycerides (mmol/L)	8.7 (0.8–17.7)	7.5 (4.8–9.3)	3.6 (1.8–8.2)	1.4 (0.6–2.1)	4.3 (1.2–15.2)	1.2 (0.8–13.0)	2.3 (1.0–5.6)
Cholesterol (mmol/L)	5.98 (4.7–8.6)	5.98 (2.9–7.7)	5.00 (3.9–5.4)	4.64 (3.2–5.7)	3.73 (3.0–5.8)	3.50 (2.8–6.6)	6.37 (4.8–9.1)
Liver adenoma(s)	Yes	Yes	No	No	No	Yes	Yes
Nephropathy	Yes	No	No	No	No	No	No
Bone disease							
*Osteopenia*	No	NR	NR	NR	NR	NR	NR
*Osteoporosis*	LS, PF	NR	NR	NR	NR	NR	NR

Legend: Biochemical parameters are presented as median and range. Legend: ^e^, significantly different compared to case E, etc.; ^, height outside of target range; LS, lumbar spine; NR, not recorded; PF, proximal femur; R, radius. Differences were considered statistically significant at *p* < 0.05

Patient A presented clinically with severe hypoglycemia’s in the first days of life, when plasma TG concentrations were 0.22 mmol/L. Enzymatic studies had confirmed diagnosis of GSD Ia, but no molecular studies had been performed at that time. She had been referred to the UMCG at the age of 16. Despite strict dietary management, height has remained below target range and she underwent a partial hepatectomy at the age of 19 years due to liver adenomas of which the largest was 5.9 cm (arrow 1 Fig. 1a). The patient was one of the very few GSD Ia patients known in the UMCG who was not growing within her target range. However, dietary compliance had been questioned over the years. In an attempt to improve her metabolic control before surgery, she was hospitalized 3 days before the procedure. Blood lactate concentrations only decreased to 2.3 mmol/L after increasing both enteral and parenteral carbohydrate intakes to supra-physiological values (4.7 and 3.9 mg/kg/min, respectively). Based on these observations, after the hospitalization the prescribed absolute dietary carbohydrate intake was increased to 5 mg/kg/min glucose, 2.2 times the estimated endogenous glucose production rate, according to literature (Huidekoper et al 2014). Following this intervention, blood lactate concentrations remained increased despite higher carbohydrate intake (ranging between 2.9 to 7.1 mmol/L). TG concentrations (absolute and CUSUM) decreased subsequentually, reflecting improved metabolic control, but she gained 8 kg of body weight, reflecting the delicate balance between under- and over-treatment. At that time, results on molecular testing became available and confirmed homozygosity for the c.79delC/p.Gln27Argfs*9 mutation in exon 1 of the *G6PC* gene, leading to a severely truncated protein without any of the essential domains necessary for the G6Pase activity (Angaroni et al 2004).

Patient B is the daughter of Turkish immigrants growing in/above the target range, (not even) adjusted for her ethnicity. She developed severe iron treatment resistant anemia due to multiple liver adenomas, for which she underwent a liver transplantation at the age of 19 years (arrow 1 in Fig. 1b). In the CUSUM-analysis, this is visible as a rapid decrease of the CUSUM, corresponding to the TG mean. This represents improved metabolic control.

The family history of patient C (family III) will be summarized in the following section. After the moment this patient, first believed to have GSD IX, received the correct diagnosis of GSD Ia (arrow 1 in Fig. 1c), dietary management and the compliance with this dietary management improved. TG values (absolute and CUSUM) subsequently normalized. In the CUSUM-analysis, this is visualized since the CUSUM decreased to 0 mmol/L, corresponding to the TG mean.

Patient D presented clinically during a gastro-enteritis at the age of 22 months with failure to thrive and hepatomegaly. After introduction of dietary management, biometrical data, liver size, and biochemical parameters of metabolic control have been outstanding. In the CUSUM-analysis, it can be seen that the CUSUM is relatively low compared to patients A, B, and C, with a maximum of 146 mmol/L depicted at the right y-axis. She is currently still on continuous nocturnal gastric drip feeding with a daily carbohydrate intake of 3.7 mg/kg/min (1.2 times the estimated endogenous glucose production) (Huidekoper et al 2014).

### Differences between patients within GSD Ia families carrying identical G6PC genotypes

Table 3 presents the clinical and biochemical parameters and long-term complications between siblings in four GSD Ia families. Heterogeneity between these GSD Ia patients is illustrated by significant differences in clinical parameters (i.e., height ranges from −2.7 to +1.9 SDS), biochemical parameters (i.e., TG_median_ ranges from 2.6 to 38.8 mmol/L), and development of long-term complications in every family.

**Table 3:** Clinical and biochemical parameters in eight GSD Ia patients from four families of whom 1–3 are followed in the UMCG and 4 is followed in the UF

Family	I		II		III				IV	
*G6PC* mutation										
*cDNA*	c.1039C > T|c.809G > T		c.900delA|c.172_173delGG		c.467G > T				c.247C > T	
*protein*	p.G270 V|p.Q347X		p.300X|p59X		p.W156 L				p.R83C	
Descent	Caucasian		Caucasian		Caucasian				Caucasian	
Case	1	2	3	4	5	6	7	8	9	10
Gender	Male	Male	Male	Female	Male	Male	Male	Male	Male	Female
Year of birth	1973	1973	1973	1976	1982	1986	1992	1997	1996	1996
Last height										
(cm)	165	176	197	166	174	181	176	174	182	179
(SDS)	−2.7^	−1.1	+1.9	−0.7	−1.4	−0.4	−1.1	−1.2	0.7	0.3
BMI										
(kg/m2)	22.1	28.9	21.7	23.6	20.4	20.1	19.7	20.1	30.0	29.4
(SDS)	+0.3	+2.4	+0.1	+0.8	−0.6	−0.7	−0.9	−0.2	NR	NR
Lactate (mmol/L)	3.7 (2.2–4.4)	3.4 (1.8–6.4)	3.3 (1.6–8.5)	5.6* (3.0–11.2)	2.6 (1.5–3.8)	2.6 (2.0–4.7)	2.7 (0.8–5.5)	2.3 (1.7–4.6)	2.6 (0.6–7.1)	2.4 (0.9–8.4)
Uric acid (mmol/L)	0.24 (0.20–0.40)	0.33* (0.25–0.43)	0.32 (0.23–0.53)	0.36* (0.23–0.60)	0.36 (0.17–0.50)	0.26 (0.14–0.51)	0.26 (0.16–0.38)	0.35 (0.15–0.63)	0.31 (0.19–0.45)	0.26 (0.20–0.42)
Triglycerides (mmol/L)	38.8 (2.5–109.9)	12.8 (2.8–15.7)	5.3 (1.9–10.3)	4.1 (2.6–9.2)	4.7 (2.7–8.4)	3.1 (0.9–5.6)	4.2 (2.1–8.6)	2.6 (1.5–5.4)	4.9 (2.1–11.6)	4.4 (2.1–13.3)
Cholesterol (mmol/L)	15.1 (4.4–42.4)	7.7 (5.2–10.7)	5.0 (3.1–6.3)	5.1 (3.8–6.5)	6.5 (4.9–8.6)	5.2 (2.9–6.4)	5.9 (3.3–7.6)	4.9 (2.7–6.2)	5.1 (4.2–6.7)	5.5 (4.2–7.3)
Liver adenoma(s)	Yes	No	Yes	Yes	Yes	No	No	No	Yes	Yes
Nephropathy	Yes	No	No	No	No	No	No	No	No	Yes
Bone disease										
*Osteopenia*	No	PF	LS, PF, R	R	No	LS, PF, R	LS	PF	TB	NR
*Osteoporosis*	LS, PF	LS	No	LS, PF	LS, PF	No	No	LS, R	No	NR

Biochemical parameters are presented as median and range. *, significantly different compared to sibling; ^5^, significantly different compared to case 5, etc.; ^, height outside of target range; NR, not recorded; LS, lumbar spine; PF, proximal femur; R, radius; TB, total body. Differences were considered statistically significant at *p* < 0.05

In family I, patient 1 was additionally diagnosed with lipoprotein lipase deficiency, but his brother was not. Patient 1 additionally developed liver adenomas and nephropathy, in contrast to his brother.

Family II was reported previously (Rake et al 2000b). The patients differ with respect to lactate, TG and uric acid concentrations. Both patients developed liver adenomas, but only patient 4 developed osteoporosis.

Family III represents four affected male GSD Ia patients, including patient C. The patients have been considered GSD type IX patients for most of their lives because of their family history suggesting X-linked inheritance and their relatively mild fasting intolerances. The latter was reflected by the fact that patient 6 from family III was the index patient with an older affected brother diagnosed after him. The brothers were initially prescribed relatively low doses of uncooked cornstarch (UCCS) during the day, and late evening meals. Surprisingly, after next generation sequencing analysis became available, it demonstrated homozygosity for the c.467G > T/p.Trp156Leu *G6PC* mutation in exon 4, known to be associated with retained residual G6Pase activity (Shieh et al 2001; Kirk et al 2013). After revision of the diagnosis, they were prescribed late-evening doses of extended release cornstarch, aiming at normalization of laboratory parameters, although dietary compliance had been limited. There were no significant differences in clinical or biochemical parameters between the family members. However, patient 5 was the only sibling that developed three liver adenomas. These have not increased in size in the subsequent 2 years.

In family IV, the siblings are identical twins. Their clinical and biochemical parameters do not differ significantly and patients 9 and 10 both developed liver adenomas. However, in contrast with this brother, at the age of 17, the liver adenomas in patient 10 developed so rapidly that liver transplantation was deemed necessary. At this age, this patient also developed nephropathy.

## Discussion

This is the first report of large heterogeneity between GSD Ia patients based on retrospective study of longitudinal clinical and laboratory data. This report shows that there are differences GSD Ia patients with homozygosity for different *G6PC* mutations and differences between patients within GSD Ia families carrying identical *G6PC* genotypes.

Based on the genotype of the patients in this study, one can speculate on the cause for the heterogeneity. In this study, patients with homozygosity for either severe nonsense mutations or active site *G6PC* mutations appear to be more severely affected clinically (i.e., patient A, B, E, and F in Tables 1 and 2). Historically, GSD Ia diagnosis required the confirmation of impaired G6Pase enzyme activity in frozen liver tissue. Nowadays genetic testing (including *G6PC* gene sequencing) is the preferred method since it is less invasive. Based on in vitro studies, many *G6PC* mutations can be categorized according to their predicted catalytic, helical, or non-helical locations in the enzyme (Shieh et al 2001; Chou and Mansfield 2008; Bruni et al 1999). Genotype-phenotype correlations have not been studied systematically and are complex because by far most GSD Ia patients are compound heterozygous for different *G6PC* mutations (Bali et al n.d.; Rake et al 2002b; Wang et al 2011).

Furthermore, the differences between affected siblings with identical *G6PC* mutations suggest a contribution of additional (genetic and/or environmental) modifying factors that theoretically modify the GSD Ia phenotype.

Variations of residual endogenous glucose production may be a modifying factor in GSD Ia patients. In healthy subjects, endogenous glucose production rate is age dependent and decreases relatively with body weight and age (Huidekoper et al 2014; Bier et al 1977). Interestingly, in GSD Ia patients, whole body in vivo endogenous glucose production may reach ∼60% of normal, despite severely reduced or absent in vitro hepatic G6Pase activity (Huidekoper et al 2014; Kalhan et al 1982; Tsalikian et al 1984; Schwenk and Haymond 1986; Roden et al 2007). The origin of this glucose production is still a matter of debate. The metabolic block may be compensated for by (combinations of) residual G6Pase activity, (muscle) glucose-6-phosphatase-β, and/or alternative glycogenolysis (by the α-glucosidase or debranching pathway). Besides the product (i.e., glucose) deficiency, there is substrate (i.e., G6P) accumulation in the endoplasmic reticulum of GSD Ia patients (Bali et al n.d.; Froissart et al 2011). G6P accumulation affects transcription and enzyme activity (including carbohydrate response element binding protein and 11β-hydroxysteroid dehydrogenase) of several metabolic pathways such as glycolysis, de novo lipogenesis, and the pentose phosphate pathway, which together create the complex clinical and biochemical GSD Ia phenotype (Oosterveer and Schoonjans 2014; Melis et al 2015).

This study introduces CUSUM-analysis to visualize subtle time-dependent variations of retrospectively collected TG-concentrations in cases A-D. However, it needs to be mentioned that CUSUM-analysis of retrospectively collected TG concentrations has been complex, because time intervals between measurements were not constant. Moreover, the variations in plasma TG concentrations in GSD Ia patients are not as fast as changes in glucose concentrations in these patients. Therefore, we hypothesize that prospective application of CUSUM-analysis may be a powerful tool to identify early and critical biochemical variations in patients with inherited metabolic diseases. The correlation between CUSUM-analysis of relevant biomarkers and clinically relevant outcome parameters deserves future prospective study.

There is no clear definition of ‘good metabolic control’ for GSD Ia patients, although several biomedical targets (including growth, liver size, and standard laboratory parameters such lactate, TG, cholesterol, and uric acid levels) are mentioned in GSD I management guidelines (Rake et al 2002a; Kishnani et al 2014). TG concentrations are considered as an important biometrical parameter of metabolic control. ESGSDI has recommended to aim at TG < 6.0 mmol/L (Rake et al 2002a, b). Significant differences in adenoma development/progression have been reported between GSD Ia patients with 5-year mean TG concentrations <500 mg/dL (i.e., 5.7 mmol/L) and >500 mg/dL (Wang et al 2011). In the above mentioned reports, GSD Ia patients were considered a homogenous group (Rake et al 2002a; Kishnani et al 2014). This study emphasizes that dietary management of GSD Ia patients requires individualized approaches.

## Conclusion

We report large heterogeneity of (long-term) clinical and biochemical parameters between GSD Ia patients. Differences between patients carrying homozygous *G6PC* mutations indicate that the *G6PC* genotype is an important determinant of the phenotype. Differences between affected siblings with identical *G6PC mutations* suggest a contribution of additional (genetic and/or environmental) modifying factors to GSD Ia symptoms and signs. CUSUM analysis can be helpful to identify early changes in metabolic control for individual patients, which opens up possibilities to move toward precision medicine for metabolic patients.

BMI, body mass index; CGM, continuous glucose monitoring; CUSUM, cumulative sum; ESGSDI, European Study on Glycogen Storage Disease Type I; G6P, glucose-6-phosphate; G6Pase, glucose-6-phosphatase; *G6PC*, glucose-6-phosphatase, catalytic subunit; LS, lumbar spine; PF, proximal femur; R, radius; TG, triglycerides; UCCS, uncooked cornstarch.
